# Multicentre evaluation of perinatal pharmacological management in women with bipolar disorder

**DOI:** 10.1192/j.eurpsy.2021.240

**Published:** 2021-08-13

**Authors:** M. Casanova Dias, I. Jones, A. Wieck

**Affiliations:** 1 National Centre For Mental Health, School Of Medicine, Cardiff University, Cardiff, United Kingdom; 2 Greater Manchester Mental Health Foundation Trust, University of Manchester, Manchester, United Kingdom

**Keywords:** bipolar disorder, pregnancy, Postpartum, perinatal

## Abstract

**Introduction:**

The pharmacological management of women with bipolar disorder in the perinatal period is challenging. This population has a high recurrence rate, but some medications can be a concern in pregnancy and breastfeeding. Little is known about prescribing practices in perinatal services, and the impact of medication on recurrence rates.

**Objectives:**

To describe 1. the use of medication in women with bipolar disorder in the perinatal period and 2. the impact of medication on the rate of recurrence.

**Methods:**

Clinical data was collected from pregnant women with diagnosis of bipolar disorder in the nine participating centres and who were not experiencing an episode of illness entering the postpartum period. Data were analysed for association using χ^2^ tests and logistic regression.

**Results:**

In this sample of 167 women, 55% were taking medication at delivery: 37% antipsychotics, 15% mood stabilisers, 25% antidepressants. In 12 cases medication was reduced before delivery. 42% experienced a recurrence, with 30% being a manic/psychotic episode. There was no significant association between taking medication and recurrence c^2^(1)=0.72, p=0.79. There continued to be no association when adjusted for severity (previous admissions, age at first treatment, bipolar subtype) and type of medication OR 0.57 95%CI [0.08; 4.29], p=0.59.

**Conclusions:**

A high number of bipolar women are taking medication before delivery and in the majority antipsychotics are prescribed. The postnatal recurrence rate in both medicated and unmedicated women is high. Further work is needed in larger samples to provide clinical guidance for women and their clinicians.

**Disclosure:**

No significant relationships.

